# The societal costs of problem gambling in Sweden

**DOI:** 10.1186/s12889-020-10008-9

**Published:** 2020-12-18

**Authors:** T. Hofmarcher, U. Romild, J. Spångberg, U. Persson, A. Håkansson

**Affiliations:** 1 IHE - The Swedish Institute for Health Economics, Box 2127, 22002 Lund, Sweden; 2 The Public Health Agency of Sweden, Box 505, 831 26 Östersund, Sweden; 3 Clinical Addiction Research Unit, Lund University, Malmö Addiction Center, 205 02 Malmö, Sweden

**Keywords:** Gambling, Societal costs, Cost-of-illness, Economic burden, Sweden

## Abstract

**Background:**

Problem gambling is a public health issue affecting both the gamblers, their families, their employers, and society as a whole. Recent law changes in Sweden oblige local and regional health authorities to invest more in prevention and treatment of problem gambling. The economic consequences of gambling, and thereby the potential economic consequences of policy changes in the area, are unknown, as the cost of problem gambling to society has remained largely unexplored in Sweden and similar settings.

**Methods:**

A prevalence-based cost-of-illness study for Sweden for the year 2018 was conducted. A societal approach was chosen in order to include direct costs (such as health care and legal costs), indirect costs (such as lost productivity due to unemployment), and intangible costs (such as reduced quality of life due to emotional distress). Costs were estimated by combining epidemiological and unit cost data.

**Results:**

The societal costs of problem gambling amounted to 1.42 billion euros in 2018, corresponding to 0.30% of the gross domestic product. Direct costs accounted only for 13% of the total costs. Indirect costs accounted for more than half (59%) of the total costs, while intangible costs accounted for 28%. The societal costs were more than twice as high as the tax revenue from gambling in 2018. Direct and indirect costs of problem gambling combined amounted to one third of the equivalent costs of smoking and one sixth of the costs of alcohol consumption in Sweden.

**Conclusions:**

Problem gambling is increasingly recognized as a public health issue. The societal costs of it are not negligible, also in relation to major public health issues of an addictive nature such as smoking and alcohol consumption. Direct costs for prevention and treatment are very low. A stronger focus on prevention and treatment might help to reduce many of the very high indirect and intangible costs in the future.

**Supplementary Information:**

The online version contains supplementary material available at 10.1186/s12889-020-10008-9.

## Background

Gambling problems are a widespread phenomenon in many countries around the world. A recent review of prevalence rates across all continents found that adult past-year problem gambling rates ranged from 0.1 to 5.8% in the period 2000–2015 [1]. Country-specific estimates in Europe ranged from 0.1 to 3.4%, although different methodological procedures and instruments limit comparisons. These estimates are also in line with an older review of prevalence rates in Europe [2]. In Sweden, 1.3% of the population aged 16–87 experienced gambling problems according to the latest estimate from 2018, and an additional 2.9% experienced less serious sub-clinical problems [3].

Gambling problems cause harm not only to individuals with gambling problems, but also to their families, their employers, and society as a whole. Harms from gambling comprise financial, social and health problems [4, 5]. This has been confirmed through both clinical and epidemiological studies [6–8]. Gamblers may use gambling to escape everyday problems and psychological distress and may view gambling as a means to solve their aggravated financial situation [9–11]. Too much time and money spent on gambling can lead to legal problems, family and relationship problems, and job loss. Mental health problems, such as depression and anxiety, harmful use of alcohol, violent behavior, and even suicide are additional consequences; nationwide register data from Sweden demonstrated a 15-fold increase in suicide mortality in individuals who had received a gambling disorder diagnosis in the health care system [12, 13]. Studies have shown that problem gambling may have severe implications for the workplace [14]. The harms for the workplaces vary from decrease in productivity to embezzlement [15, 16]. Concerned significant others (CSOs) are also affected by gambling problems. Harms range from mental and physical health problems to financial problems [17, 18]. In addition, intimate partner violence (IPV) is associated with problem gambling. Meta-analyses revealed that over one third of problem gamblers report being victims of physical IPV or perpetrators of physical IPV and that the prevalence of problem gambling in IPV perpetrators is 11.3% [19]. Many times, both the gamblers and their families experience feelings of stigma, guilt, and shame. In an earlier Swedish study, as many as 18.2% of the population were considered CSOs and they reported more negative life events in a 1-year follow-up study [20].

From a clinical perspective, a watershed event was the inclusion of serious gambling problems as a medical condition (‘pathological gambling’) in the third edition of the American Psychiatric Association’s Diagnostic and Statistical Manual of Mental Disorders (APA DSM-III) in 1980 [21]. In APA DSM-5, adopted in 2013, the condition was renamed to gambling disorder and it was re-classified from the group of impulse-control disorders to the group of substance-related and addictive disorders [22]. This re-classification put gambling disorders in the same group as alcohol use disorder and tobacco use disorder along with other substance use disorders. In contrast mainly to the fields of alcohol and tobacco, gambling problems are underexplored [23]. Access to gambling has also become easier during the last two decades through online gambling. While alcohol and tobacco restrictions have featured high on the agenda of health policy makers in recent decades, such as the WHO Framework Convention on Tobacco Control [24], gambling often flies under their radar. Generally, international framework conventions on gambling are lacking. An exception is a recommendation by the European Commission issued in 2014 on protecting consumers and children from online gambling [25].

In order for stakeholders to take adequate policy measures against problem gambling, it is of great relevance to have a good understanding of the prevalence, risk factors, and therapeutic interventions. In addition, an understanding of the full costs imposed on society is crucial, in order to weigh the costs and benefits of interventions against each other.

A handful of studies have assessed the societal costs of problem gambling. An early study from Australia, which several subsequent international studies attempted to emulate, found that the costs amounted to 1.8–5.6 billion Australian dollars (AUD) for the years 1997–98, corresponding to 0.3–1.0% of gross domestic product (GDP) [26]. A follow-up study for the years 2008–09 put the costs to AUD 4.7–8.4 billion per year, corresponding to 0.4–0.7% of GDP [27]. Studies for different states in the United States [28], Alberta in Canada [29], Wales in the United Kingdom [30], and the Czech Republic [31] have also found great costs to society. However, a direct comparison of the study results is difficult due to a range of methodological differences [32, 33]. These include decisions regarding which costs included or excluded (a choice often driven by data availability), how included cost types are valued, and double counting of costs. Another issue is insufficient evidence on whether observed relationships between problem gambling and personal circumstances (mental health problems, unemployment, etc.) are causal or correlational. All of these factors impede the transferability of the study results to other countries.

The purpose of this study is to carry out a cost-of-illness analysis of the societal costs of problem gambling in Sweden in 2018. No other analysis of the costs of problem gambling has been conducted for any Nordic country. An additional motivating factor for this study are recent law changes in Sweden, which came into force on January 1, 2018. While this condition was not covered by these laws prior to 2018, these recent law changes [12] oblige regional health care providers and local social care providers to invest in prevention and treatment of gambling problems. This is likely to have implications for the current and future societal costs of problem gambling.

## Methods

### Study design

A cost-of-illness (COI) study was conducted to estimate the societal costs of problem gambling in Sweden. There are two methods for estimating costs in COI studies, the prevalence method and the incidence method [34]. The prevalence method is the most common one and is also used in this study. It entails the estimation of costs incurred during a given year. In this study, the reference year is 2018. A prevalence-based COI study proceeds in several steps. First, the study population needs to be defined, i.e. the number of people with gambling problems and other affected entities. Second, the types of costs to be included need to be defined. Third, the calculation of all costs needs to be defined. The latter also includes methods to address cases of relationships with an unclear direction of causality.

### Study population

Gambling problems affect not only the gamblers but also family members and employers, victims of crimes committed by gamblers, and society as a whole. In this study, information on the number of people with gambling problems is obtained from the Swedish Longitudinal Gambling Study (Swelogs), conducted by the Public Health Agency of Sweden [35]. Swelogs is a representative survey of the Swedish adult population (aged 16–87) and was last conducted in 2018 with an invited sample size of 13,251 people (around 0.2% of the adult population) and a response rate of 38%.

The results from the Swelogs survey can be used to categorize all respondents into four categories (non-problem gamblers, low-risk gamblers, moderate-risk gamblers, problem gamblers) based on the Problem Gambling Severity Index (PGSI), a validated index system [36]. The definition of problem gambling has varied throughout the literature, and international comparisons of problem gambling rates have referred to measures based on different diagnostic or screening tools [1], including the PGSI. The group classifications derived from the PGSI are based on nine items, which include core symptoms of problem gambling, such as, for example, gambling for more than one can afford, increased tolerance, ‘chasing losses’ behavior, health consequences of gambling, and financial consequences [36].

Table 1 provides an overview of past-year problem gambling rates and the corresponding number of people. Non-problem gamblers accounted for 95.8% of the population, while the remaining share was composed of low-risk gamblers (2.9%), moderate-risk gamblers (0.7%), and problem gamblers (0.6%). The Swelogs survey also provides information on the number of people living in the same household as the gambler (see Table 1), as well as information on the gambler’s health, relationship status, employment, and quality of life (see Additional file 1: Table A1 in the Online appendix).

**Table 1:** Past-year problem gambling in Sweden in 2018, number of affected individuals. Number and percentage of adults with low-risk, moderate-risk and problem gambling, and estimated number of other individuals living in their households. Results are based on the Swelogs survey. The adult population is 16–87 years. Low-risk gamblers have a PGSI index of 1–2, moderate-risk gamblers of 3–7, and problem gamblers of 8–27

	Share of adult population	Number of people	Number of other people living in the same household
Low-risk gamblers	2.9%	236,000	410,000
Moderate-risk gamblers	0.7%	56,000	99,000
Problem gamblers	0.6%	45,000	66,000

### Types of costs

A societal perspective was chosen to define the costs of problem gambling. Previous studies and reports on the costs of problem gambling were searched in order to identify all relevant types of costs [26–31, 37, 38]. The compiled list of potential types of costs was then complemented to ensure that the included cost types reflect national circumstances. Table 2 shows all types of costs that have been considered relevant in the calculation of the societal costs of problem gambling in Sweden, irrespective of whether it was possible to quantify the costs in the next steps.

**Table 2:** Types of costs of problem gambling in Sweden. Types of costs and their causality adjustment factors (%). A factor of 20% means that total costs were multiplied by 0.8 to discount costs by 20% in cases of an unclear causality

Type	Description	Causality adjustment factor*
A. Direct costs		
A.1. Treatment and care		
Treatment of gambling problems	Public and private costs for treatment and work by non-profit organizations	0%
Treatment of consequences of gambling problems	Public and private costs for treating mental illnesses and suicide attempts	20%/50%
A.2. Debt counseling and management		
Debt counseling	Costs for debt counseling	20%
Debt management	Costs for debt recovery, debt restructuring, and personal bankruptcy	20%
A.3. Crime and legal costs		
Police	Costs for investigations of crimes	20%
Courts	Costs for criminal cases in general courts and social insurance-related cases in administrative courts	20%
Prisons	Costs for incarceration	20%
A.4. Prevention, research, and regulation		
Prevention	Costs for prevention of gambling problems	0%
Research	Costs for research on gambling problems	0%
Regulation	Costs for regulation and supervision of the gambling market	93%
A.5. Other direct costs		
Divorce	Costs for divorce applications	50%
Recruitment	Recruitment costs of employers	50%
Homelessness	Costs for homeless shelters	–
B. Indirect costs		
B.1. Reduced workplace productivity	Productivity loss from reduced workplace productivity	20%
B.2. Absence from work	Productivity loss from sick leave and from incarceration	20%
B.3. Unemployment	Productivity loss from unemployment	50%
B.4. Premature death	Productivity loss from suicides	20%
C. Intangible costs		
C.1. Physical violence	Reduced quality of life of the gambler and other household members from physical violence	20%
C.2. Emotional distress	Reduced quality of life of the gambler and other household members from mental illness, suicide attempts, separation, and unemployment	20%/50%
C.3. Harm to crime victims	Reduced quality of life and damage to victims from crime exposure	20%

The costs in Table 2 are grouped according to the three types of societal costs generally included in COI studies [34]. The valuation of the costs varies for every type. First, direct costs correspond to all medical and non-medical resources used in relation to gambling problems and their consequences [39]. These costs were valued based on prevailing (market) prices in this study.

Second, indirect costs correspond to productivity loss due to morbidity and mortality caused by gambling problems [39]. Costs refer here to the value of resources that are not being created due to unemployment, sick leave, and the like. The fact that individuals’ time is a limited resource for which there is an alternative cost is widely accepted in economic theory [40]. One hour of lost production thus corresponds to the value of the work carried out, defined in this study as the average gross salary plus social security contributions. Transfer payments within the social security system (unemployment benefits, sickness benefit, etc.) were not included to avoid double counting of costs. Productivity loss due to death of people of working age (defined as 16–74 years) was calculated as the present value of future earnings losses throughout the remainder of a person’s working life in this study, in line with the human-capital method commonly used in the literature [41]. Adjustments for age-specific employment rates, earnings, and background mortality were made in the calculation of future earnings losses and a real earnings growth of 2% as well as a discount rate of 3% was applied.

Third, intangible costs refer to a valuation of reduced quality of life due to gambling problems. Unlike direct and indirect costs, intangible costs have no direct connection to the use or lack of production of resources. COI studies often exclude intangible costs, as they cannot be valued with existing (market) prices. However, omitting intangible costs solely due to difficulties in valuation is not satisfactory, as the implicit assumption would be that the economic value of quality of life is zero [42]. In line with previous studies [26, 37], experiences of physical violence and emotional distress were valued based on the average compensation payment for crime victims by the Swedish Crime Victim Compensation and Support Authority.

### Calculation of costs

The size of each defined type of cost was calculated in one of two ways. The first way was to use a lump sum of the total costs that gambling problems cause. Examples are measures for prevention by different organization or earmarked research grants. The second way was a bottom-up approach in which the number of affected gamblers is multiplied with an average unit cost per person. Most of the costs were calculated in the latter way by combining epidemiological data from the Swelogs survey with unit cost data from Statistics Sweden, other authorities, and health care providers. For the calculation of crime and legal costs, we had to rely on data on the incidence of criminal activities from a study in Australia [37], which was the only recent study we identified to classify gamblers according to the PGSI system (see also Additional file 1: Table A1 in the Online appendix).

A challenge in accurately estimating the size of each type of cost is the lack of information on causal relationships. Gambling problems may not be the cause but rather the result of life circumstances (such as unemployment and divorce) or disorders (such as anxiety and depression). Previous studies have attempted to address this problem by discounting costs with a ‘causality adjustment factor’. Most studies have followed the method utilized in the 1999 report by the Australian Productivity Commission [26]. Based on expert opinions, this report assumed that around 80% of problem gamblers would still have faced similar personal and family-related consequences in the absence of gambling problems. Despite progress in understanding the nature of gambling problems since this report was published, we also discounted many costs with 20%; see Table 2. In cases where there is no or very thin evidence on the direction of causality and where it could plausibly run equally in both ways, we discounted costs with 50%; see Table 2.

All costs are presented in euros (€) and 2018 prices. The average exchange rate in 2018 was 1 euro = 10.2583 Swedish krona, and prices were adjusted to 2018 price levels using the national consumer price index when necessary.

Analyses were based on data already available from the Swelogs study, based on available ethics permission (file number 2015/1145–31/5). No further application procedure was required for the conduct of the present study.

## Results

**Fig. 1:**
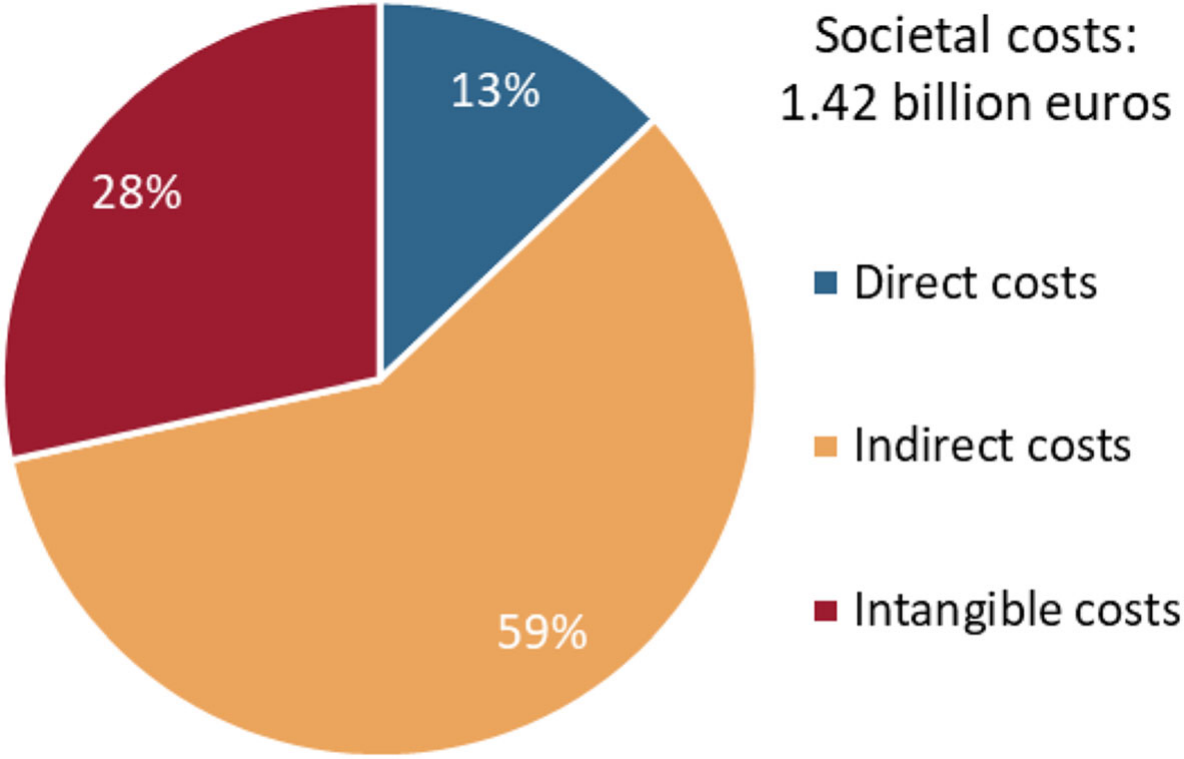
Societal costs of problem gambling in Sweden in 2018

From a societal perspective, the costs of problem gambling are composed of direct costs, indirect costs, and intangible costs. Based on the classification of cost types in Table 2, the estimated results are listed in Table 3. The results show that the societal costs of problem gambling amounted to €1419 million in Sweden in 2018. Direct costs accounted for €184 million, corresponding to 13% of the total costs; see also Fig. 1. Indirect costs accounted for €832 million and represent more than half (59%) of the total costs. Intangible costs accounted for €403 million, corresponding to 28% of the total costs.

**Table 3:** Estimated societal costs of problem gambling in Sweden in 2018 (million €). Sums do not sum up due to rounding

Type of cost	Estimated costs (million €)
A. Direct costs	
A.1. Treatment and care	
Treatment of gambling problems	3.82
Treatment of consequences of gambling problems	71.90
A.2. Debt counseling and management	
Debt counseling	1.61
Debt management	6.10
A.3. Crime and legal costs	
Police	8.57
Courts	1.06
Prisons	2.13
A.4. Prevention, research, and regulation	
Prevention	1.27
Research	1.24
Regulation	0.42
A.5. Other direct costs	
Divorce	0.24
Recruitment	85.56
Homelessness	n.e.
B. Indirect costs	
B.1. Reduced workplace productivity	70.64
B.2. Absence from work	0.62
B.3. Unemployment	641.70
B.4. Premature death	119.14
C. Intangible costs	
C.1. Physical violence	
Gambler	23.58
Household members	38.58
C.2. Emotional distress	
Gambler	116.33
Household members	196.95
C.3. Harm to crime victims	27.95
Sum of costs	1419.42

*N. e*. = not estimated

### Direct costs

#### Treatment and care

##### Treatment of gambling problems

Treatment of people with gambling problems is carried out by publicly funded providers and by non-profit organizations. Health care providers and social care providers are obliged to offer support and treatment to people with gambling problems and to prevent gambling problems since January 1, 2018. The National Board of Health and Welfare recommends these providers to offer cognitive behavioral therapy (CBT) with a focus on gambling and also motivational interviewing in certain circumstances. There is no comprehensive information on the number of people who have sought and received help by these providers in 2018, but the Ministry of Health and Social Affairs previously estimated that 10% of problem gamblers would seek help. This corresponds to around 4500 people, who receive on average 9 sessions of treatment. We assumed 2 h of work per session for a psychiatric nurse or a social worker and individual treatment, as it is difficult to gather enough people for group therapy. The average hourly labor cost of a psychiatric nurse was €31 (including social security contributions) and €29 of a social worker in 2018. Assuming that half of all patients are treated by psychiatric nurses and the other half by social workers results in treatment costs of €2.44 million. Only patients treated by health care providers have to pay an out-of-pocket patient fee (around €19) for every session. The total cost of out-of-pocket patient fees was €0.24 million, as there was a state-regulated annual high-cost threshold of €107 per patient on these fees in 2018 which prevents patients from paying fees in excess of the threshold on any outpatient services. No drug treatment is recommended by the National Board of Health and Welfare and hence there are no such costs included, even though some prescription of the drug naltrexone might occur in clinical practice.

There is a nationwide publicly funded support line that people with gambling problems and their relatives can call to receive advice and support. The support line is run by the Centre for Psychiatry Research at the Karolinska Institute and costed €0.49 million. In addition, the Centre for Psychiatry Research developed an online training course on treating gambling problems in 2018, which cost €0.02 million.

Non-profit organizations are an important complement to publicly funded providers of treatment. These organizations are often run by former problem gamblers and their relatives. They offer treatment in the form of advice and support, but also work with prevention and dissemination of information about the causes and consequences of gambling problems. There are no detailed records on the extent of the activities of all non-profit organizations. As an approximation of the value of their work done, we use the total amount of public grants received by the organizations. The public grants by the Public Health Agency of Sweden amounted to €0.63 million in 2018.

##### Treatment of consequences of gambling problems

People with gambling problems often suffer from several mental illnesses [12]. Data from the Swelogs survey showed that a greater share of people with gambling problems experienced poor mental health (measured with the Kessler Psychological Distress Scale - K6) than in the general population. To estimate the number of people seeking care for mental illness caused by gambling problems, we subtracted the share of people with poor mental health in the general population from the share observed in people with gambling problems. This yields an excess share of poor mental health, half of which was assumed to be caused by gambling problems. This results in around 30,800 people with mental illnesses caused by gambling problems. However, not all people with mental illness seek treatment. Based on the propensity to seek care for mental illness at health care providers in the general population (62%) observed in a previous study [43], around 19,000 people with gambling problems could have been expected to seek care. The average annual cost of treating mental illness (defined as the average of costs of bipolar disorder, depression, and generalized anxiety disorder) was around €3500 per patient according to a Swedish registry study [44]. This results in total costs of €67.29 million for treating mental illnesses. Patients also have to pay an out-of-pocket patient fee (around €19) for every treatment session. As above, we assume that all patients reached the annual high-cost threshold on these fees, resulting in a total private cost of €2.04 million.

People with gambling problems are at an increased risk of suicide. A Swedish registry study showed that the risk of death by suicide was 15.1 times higher among problem gamblers than in the general population [13]. While completed suicides constitute an indirect cost, suicide attempts leading to medical treatment constitute a direct cost. We assumed that the increased risk of completed suicide also applies to suicide attempts. Based on the rate of suicide attempts requiring hospital care in the general population (109 attempts per 100,000 inhabitants), the number of problem gamblers, and a causality adjustment of 20%, the estimated number of attempts was around 590. The average hospital treatment cost for an attempt was €4300, resulting in a total cost of €2.55 million. Patients also have to pay an out-of-pocket patient fee (around €10) for every day at the hospital. Given an average care time of 3.3 days, the total private cost was €0.02 million.

#### Debt counseling and management

##### Debt counseling

Gambling problems can lead to a difficult financial situation when the money lost on gambling consistently exceeds one’s income. People with financial problems can turn to debt counseling, which is offered by all municipalities. More than 18,000 people received debt counseling in 2018 and 14% of all cases were related to gambling problems according to the Swedish Agency for Public Management. There were 258 (full-time equivalent) case workers working with debt counseling in 2018 and the average annual labor cost amounted to €55,900 per case worker. The staff cost for gambling problem-related debt counseling thus amounted to €1.61 million, after a causality adjustment of 20%.

##### Debt management

When people with gambling problems start to become indebted, costs for the Swedish Enforcement Authority arise for processing applications by creditors and for collecting debts. The Enforcement Authority also processes applications for debt restructuring and for personal bankruptcy. In 2018, the Enforcement Authority dealt with around 413,900 debtors in connection with enforcement and debt recovery. Even though the average number of cases per debtor was around five, we only assume one case per debtor. The average cost per case was €71. Around 25,000 people applied for debt restructuring and the average cost per case was €960. Around 1200 people were subject to personal bankruptcy and the average cost per case was €740. We assumed that 14% of all cases of the Enforcement Authority were related to gambling problems, in line with debt counseling data. The total costs for gambling problem-related debt recovery, debt restructuring, and personal bankruptcy amounted to €6.10 million, after a causality adjustment of 20%.

#### Crime and legal costs

A difficult financial situation combined with poor mental health is a strong risk factor for social problems. To continue to finance their gambling activities, some people with gambling problems may commit crimes. Gambling-related crime typically consists of non-violent, income-generating offences, such as theft and shoplifting [45]. No Swedish data on the incidence of criminal activities by people with gambling problems were available, and instead, data from a recent study in Australia were used [37]. We assumed that a person who commits a crime only commits a single crime per year (which is an underestimate) and that all committed crimes are reported (which is an overestimate). This resulted in around 12,400 gambling-related crimes, after a causality adjustment of 20%. How crimes of the type typically committed by people with gambling problems (we used theft and shoplifting) are handled in the Swedish justice system was based on data from the National Council for Crime Prevention.

##### Police

Around 29% of all committed crimes relating to theft and shoplifting led to a police investigation in 2018. We assumed that only crimes actually investigated constituted a cost to the police. The average time for an investigation was 29.5 h and the cost per hour was €80 in 2018, corresponding to an average cost per investigated crime of €2400. Based on crime incidence data, the number of gambling-related crimes investigated by the police was around 3600, after a causality adjustment of 20%. This led to total costs of €8.57 million for the police.

##### Courts

Of all crimes relating to theft and shoplifting investigated by the police, around 22% led to a prosecution in district courts. We assumed that only crimes leading to prosecution constituted a cost to the courts. The average cost per court case was €1340 in 2018. Based on the estimated number of crimes investigated by the police, the number of people with gambling-related prosecutions in courts was around 800. This led to total costs of €1.06 million for the courts.

##### Prisons

Of all crimes relating to theft and shoplifting handled in the courts, around 10% led to a prison sentence. The median length of the prison sentence was 4 months. The average daily prison cost per incarcerated person was €336 in 2018. Based on the estimated number of people prosecuted in courts, the number of people with gambling-related incarcerations was around 50. This led to total costs of €2.13 million for prisons.

#### Prevention, research, and regulation

##### Prevention

The Public Health Agency of Sweden is tasked by the government to develop and disseminate support material to prevent gambling problems. Cost associated with this task amounted to €0.91 million in 2018. The 21 County Administrative Boards received a total of €0.34 million in public funding for work with prevention of gambling problems. The Swedish Sports Federation funded a website directed toward high school students about match fixing and gambling problems at a cost of €0.02 million. The total cost of prevention amounted to €1.27 million, even though some activities included in “treatment and care” (Treatment and Care) also relate to prevention, such as the nationwide support line or the work by non-profit organizations.

##### Research

The state-owned gambling company Svenska Spel supported university-based research activities with €0.88 million in 2018. A university-based research project received funding worth €0.15 million from a public research council in 2018. The Swelogs survey carried out by the Public Health Agency of Sweden at a cost of €0.21 million in 2018 was also a research-related activity. The total cost of research amounted to €1.24 million.

##### Regulation

The Swedish Consumer Agency and the Swedish Gambling Authority jointly oversee the marketing activities of gambling companies. According to the Swelogs survey, people with gambling problems say that gambling advertisements triggered them to gamble more than they had intended to. Some of the costs of regulation and supervision of the gambling market thus constitute direct costs. The Consumer Agency spent €0.05 million on activities to improve awareness of the gambling market. The Gambling Authority spent €5.81 million on control and supervision of the gambling market. The costs of regulation related to gambling problems amounted to €0.42 million, assuming that the joint total costs of the two authorities are distributed according to the share of people with gambling problems (4.2%) among all people who gambled (58%).

#### Other direct costs

##### Divorce

Divorce and separation are commonly observed among people with gambling problems, according to the Swelogs survey. Only divorce gives rise to a direct cost in the form of a mandatory application fee of around €88 to the district court. The largest costs are intangible and relate to emotional distress (Emotional distress). To estimate the number of divorces caused by gambling problems, we subtracted the share of divorces and separations in the general population from the share observed in people with gambling problems. This yields an excess share of divorces and separations, half of which was assumed to be caused by gambling problems. As no distinction between divorces and separations was made in the Swelogs survey, we further assumed that half of all cases involved divorces and the other half separations. This resulted in around 2800 divorces due to gambling problems. The total direct cost of divorces was thus €0.24 million.

##### Recruitment

When a person is dismissed or has to quit work because of gambling problems, expenses arise for the employer in conjunction with hiring a replacement. There is no direct information in the Swelogs survey on how many people have become unemployed. However, the survey contains information about a person’s main employment status (including unemployment) during the past 12 months. To estimate the number of unemployed people, we subtracted the share of unemployed people in the general population from the share observed in people with gambling problems. This yields an excess share of unemployment, half of which was assumed to be caused by gambling problems. This corresponds to 14,800 unemployed people and all of them were assumed to have become unemployed in 2018. In line with a previous study [37], we assumed that an employer’s cost of recruitment corresponds to 10% of the average annual labor cost of an employee. Given an average annual labor cost of €57,900 in Sweden in 2018, the total cost of employers for recruitment was €85.56 million.

### Indirect costs

#### Reduced workplace productivity

Reduced workplace productivity of employed people, due to gambling during working hours and gambling-caused emotional distress experienced during working hours, gives rise to a productivity loss. This type of indirect cost measures how much more work could have been done in the absence of gambling problems. The Swelogs survey contains information on the number of employed people and on whether they have been gambling during working hours. There is no information on the exact extent of gambling during working hours though. A Czech study estimated that workplace productivity was 7 to 12% lower among moderate-risk gamblers and problem gamblers than among non-problem gamblers, but no decrease was stated for low-risk gamblers [31]. We used the lower bound estimate of 7% for moderate-risk gamblers and the upper bound estimate of 12% for problem gamblers and combined it with the average labor cost of an employee. To estimate the number of affected people, we subtracted the share of people gambling during working hours in the general population from the share observed in people with gambling problems. This yields an excess share of gambling during working hours, which corresponds to 3300 moderate-risk gamblers and 8300 problem gamblers in the age group 16–74 years, after a causality adjustment of 20%. Given an average annual labor cost of €57,900 in Sweden in 2018, the total cost from reduced workplace productivity was €70.64 million.

#### Absence from work

The consequences of gambling problems, such as mental illnesses, suicide attempts, and incarceration, give rise to a productivity loss, as they force employed people to be absent from work. This type of indirect cost measures how much more work could have been done if the gambler had been present at work. The Swelogs survey does not contain any information on sick leaves that lasted shorter than half a year, while the share of people who indicated that they were on sick leave for most of the past 12 months did not differ between people with and without gambling problems. People with frequent sick leaves might eventually become unemployed and these effects are included in the calculations below (Unemployment). For people estimated to be incarcerated in the calculations above (Crime and legal costs), a productivity loss from absence from work can be calculated. However, not all of these people would have worked. We applied the average employment rate of 68.5% in Sweden in 2018 to the number of incarcerated people in the age group 16–74 years and assumed a prison sentence to last for 4 months on average. Given an average monthly labor cost of €4800 in Sweden in 2018, the cost from absence from work due to incarceration was €0.62 million.

#### Unemployment

Unemployment gives rise to a productivity loss, if gambling problems were the reason for the job loss. As part of the calculations of recruitment costs (Other direct costs), the number of unemployed people due to gambling problems was estimated to be 14,800. As these people indicated that they were unemployed for most of the past 12 months, we assumed an average length of 9 months of an unemployment spell. Given an average monthly labor cost of €4800 in Sweden in 2018, the total cost from unemployment was €641.70 million.

#### Premature death

Deaths of working age people give rise to a productivity loss equal to the expected value of their work during their remaining working life. Only premature deaths due to suicide are considered. Based on the rate of completed suicides in the general working age population (15.3 deaths per 100,000 inhabitants), an increased risk factor of 15.1 for suicides among problem gamblers in Sweden [13], the number of problem gamblers in working age, and a causality adjustment of 20%, the estimated number of suicides was 75. The median age of gambling problem-related suicide was 32.5 years in Sweden [13]. The present value of the expected future productivity loss of an average person aged 25–34 during their remaining working life amounted to €1.59 million in Sweden in 2018. Drawing on the median age of suicide, the total cost from premature death was €119.14 million.

### Intangible costs

#### Physical violence

Reduced quality of life because of physical violence gives rise to an intangible cost. An example is the (threat of) use of violence by loan sharks when the gambler struggles to repay debts. The Swelogs survey contains information on whether a person has been subjected to physical violence or the threat of physical violence making them scared during the past 12 months. To estimate the number of people exposed to violence, we subtracted the share of affected people in the general population from the share observed in people with gambling problems. This yields an excess share of exposure to violence, corresponding to around 10,500 people with gambling problems, after a causality adjustment of 20%. Even though exposure to violence could have occurred several times during the past year, only one incidence per person was assumed. Given an average crime victim compensation payment of €2250, the cost to the gambler was valued to be €23.58 million.

If a person with gambling problems is exposed to physical violence, it can also affect other people in the household, such as the gambler’s partner, their children, or the parents of adolescent gamblers. Even though the Swelogs survey does not contain information on whether other household members were affected by physical violence (or the threat of it), we assumed that all household members of gamblers exposed to physical violence were also affected. This concerned around 17,200 people and resulted in costs of €38.58 million.

#### Emotional distress

Reduced quality of life because of emotional distress gives rise to an intangible cost. Emotional distress caused by the consequences of gambling problems can be more of a long-term nature (mental illness or unemployment) or more of a short-term nature (suicide, suicide attempt, separation). In line with the calculations above, we defined events of emotional distress experienced by the gambler based on the incidence of mental illness (affecting 30,800 gamblers), suicide attempts (590 gamblers), separations (5600 gamblers), and unemployment (14,800 gamblers). We allowed for the possibility to experience all four types of distress during the past year. Given an average crime victim compensation payment of €2250, the cost to the gambler was valued to be €116.33 million.

Other people living in the same household as the gambler can also be affected by the gambler’s emotional distress. Even though the Swelogs survey does not contain information on whether other household members were affected by emotional distress, we assumed that all household members of gamblers with events of emotional distress were also affected. This resulted in around 87,700 events of emotional distress of the gambler’s household members, which resulted in costs of €196.95 million.

#### Harm to crime victims

Crimes committed by people with gambling problems cause harm and damage to the victims. Not all victims of crime must be persons. Companies can also be affected, according to how the question in the underlying Australian survey was framed. As part of the calculations of crime and legal costs (Crime and legal costs), the number of gambling-related crimes was estimated to be around 12,400. Given an average crime victim compensation payment of €2250, the cost of harm to crime victims was valued to be €27.95 million.

## Discussion

### Comparison of cost estimates

This study estimated the societal costs of problem gambling to be €1.42 billion in Sweden in 2018. These results can be compared to previous international studies, once the results are standardized by countries’ GDP. Table 4 shows that the societal costs of problem gambling corresponded to 0.30% of GDP in Sweden. The first major COI study of gambling problems conducted in Australia found that the annual costs amounted to 0.3–1.0% of GDP for the years 1997–98 [26]. A follow-up study put the annual costs to 0.4–0.7% of GDP for the years 2008–09 in Australia [27]. A more recent study conducted in the Czech Republic estimated the costs to be equal 0.37% of GDP in 2012 [31].

**Table 4:** Societal costs of problem gambling compared to other measures

Benchmark	Benchmark value in 2018	Value
Number of people with gambling problems	337,000	€4212
Number of inhabitants	10,175,00	€139
Gross domestic product	€471,196 million	0.30%
Gambling market’s net revenue	€2284 million	62%
Tax revenue from gambling	€638 million	223%
Direct and indirect costs of alcohol consumption	€5877 million^a^	17%
Direct and indirect costs of smoking	€3073 million^a^	33%

Notes: ^a^ The benchmark value for alcohol consumption is from 2017 and for smoking from 2015, and these costs are only compared with the direct and indirect costs of problem gambling. Data on people with gambling problem come from the Swelogs survey and data on inhabitants and GDP from Statistics Sweden. Data on the gambling market come from the Swedish Gambling Authority and data on tax revenue from the Swedish National Financial Management Authority

The fact that the cost estimate for Sweden in this study is at the lower end of what previous studies have found has several explanations. Costs are dependent on the prevalence of gambling problems, i.e. the higher the number of people affected, the higher the costs. In the first Australian study for the years 1997–98, people with moderate and severe gambling problems accounted for 2.1% of the adult population. This can be roughly compared to 1.3% of moderate-risk and problem gamblers in this study for Sweden. The Czech study comprised 123,000–170,000 problem gamblers and 40,000–80,000 pathological gamblers. This study for Sweden, which has about 10 million inhabitants just as the Czech Republic, comprised 56,000 moderate-risk gamblers (problem gamblers in the Czech study) and 45,000 problem gamblers (pathological gamblers in the Czech study). However, we included also low-risk gamblers, a large group of 236,000 people whose sheer numbers drove up costs.

Other explanations for country differences in the size of costs are different levels of comprehensiveness in the inclusion of cost types and their valuation. For instance, the Australian study for 1997–98 and the Czech study included intangible costs for both depression and thoughts of suicide of the gambler, whereas in this study all kinds of mental illnesses of the gambler were only included once in the intangible costs of emotional distress. These two studies also included costs for job search by the gambler, but the alternative cost of the time spent on searching jobs cannot be included twice, as this time is already captured in the costs of unemployment. Furthermore, the size of the value used to quantify intangible costs (i.e. the average compensation payment for crime victims) is very much dependent on a country’s judicial tradition.

Apart from GDP, the study results can also be compared to a range of other measures to get a better idea of ​​the size of the societal costs of problem gambling. Table 4 shows that the costs per person with (any degree of) gambling problems were over €4000. The cost per inhabitant were €139. An interesting aspect is a comparison with the gambling market. The societal costs corresponded to 62% of the gambling market’s net sales, i.e. revenues minus winnings. In addition, the societal costs amounted to more than twice the size (223%) of the tax revenue from gambling.

From a public health perspective, it is also important to compare the costs of problem gambling with costs of other public health issues of an addictive nature. Alcohol consumption and smoking are two examples of major public health issues. Previous studies of the societal costs of alcohol consumption and smoking in Sweden indicated costs of €5.88 billion and €3.07 billion, respectively [46, 47]; see Table 4. These studies did either not include intangible costs or used a different methodology to value them. Only a comparison of direct and indirect costs is therefore possible. Direct and indirect costs of problem gambling combined amounted to €1.02 billion in this study. This corresponded to around one sixth of the equivalent costs of alcohol consumption and one third of the costs of smoking in Sweden.

Direct costs are by far the smallest of the three overarching cost types in this study accounting for only 13% of the societal costs, which is similar to the results in the above-mentioned studies in Australia and the Czech Republic. Even more striking is the finding that the sum of the costs for treatment of gambling problems, debt counseling, and prevention correspond to merely 0.5% of the societal costs. This is in spite of law changes that obliged health care providers and social care providers to invest more in prevention and treatment of gambling problems as of 2018. A crucial assumption in the calculations of treatment costs was that only problem gamblers are offered CBT treatment and, more importantly, only 10% of them will actually seek treatment. In clinical practice, it is indeed difficult to find problem gamblers and make them seek treatment. Raising awareness of the availability of services that can help them is crucial. Prevention efforts in schools are also important from a long-term perspective. More resources invested in effective measures of treatment and prevention now could help to reduce many of the very high indirect and intangible costs of problem gambling in the future. Prevention of problem gambling may include supply reduction, reduction of demand and limitation of negative consequences. Systematic reviews have shown that most interventions have a low certainty of evidence. Although more research is needed, the evidence for preventive measures is so far low due to lack of research on how long-term educational programs in schools and personalized feed-back could impact gambling behavior [48]. Furthermore, the new Gambling Act in Sweden from 2019 stresses the duty of care of the gambling operators, requiring them to identify the signs of problematic gambling and intervene in order to prevent harm [49].

While this study is a cost of illness study with no estimations of the societal benefits of gambling, the costs of gambling fall on individuals, their family members, the health care sector and social care services, as well as employers. These entities are usually not the ones who receive the benefits of gambling in Sweden, as tax revenue from gambling goes into the state coffers at the national level instead of being earmarked for work by local and regional health authorities.

### Limitations

Most cost estimates in this study are directly based on the number of people with gambling problems. We drew on results from the Swelogs survey to obtain data on this. Over 13,000 people (around 0.2% of the adult population) were included in this representative survey. The response rate was 38%. Systematic differences between those who responded and not responded cannot be ruled out, even though the results were adjusted with personal calibration weights to compensate for varying selection probabilities and response bias. For instance, men and women might perceive their state of health differently and overestimate or underestimate their condition. Recall bias and dishonest responses on sensitive questions can also lead to underestimation. However, the Swelogs survey for 2018 is the most recent survey on this topic and it is a rich source of information of people’s lives. The estimated numbers are also the ones used by the Swedish authorities. In the online appendix, a scenario analysis is provided in which the number of people with gambling problems is varied according to the 95% confidence interval estimates (Additional file 2: Appendix figure A1). This results in a lower bound estimate of €1.04 billion and an upper bound estimate of €1.80 billion of the societal costs.

Another type of uncertainty in the analysis is a lack of evidence on the causal relationship between gambling problems and many different personal circumstances (mental health problems, unemployment, separation, etc.). For instance, unemployment may have been caused by gambling problems, but unemployment could equally well have led to gambling problems. The direction of causality between gambling problems and unemployment thus runs both ways. To address this type of uncertainty, many costs were discounted by a certain factor in the analysis. The choice of the exact size of this factor was partly based on previous studies but it remains somewhat arbitrary. Table 2 provides an overview of the causality adjustment factor used for each cost type. This overview also serves as a guidance for priority areas of future research to disentangle causes from consequences of gambling problems.

The comprehensiveness of the analysis in terms of inclusion and exclusion of relevant cost types clearly affects the size of the societal costs. Data availability was the key factor determining the choice to include or exclude certain cost types. A general knowledge gap was how and to what extent household members (including children) and non-household members were affected by the gambler’s circumstances. Some effects on household members were only included in the intangible costs in this study. Among the included direct costs, information on unpaid work by non-profit organizations would need to be more comprehensively assessed, which would probably drive up costs. Excluded direct costs were costs of visits to emergency psychiatry caused by suicidal thoughts or other psychiatric manifestations, health care costs related to cardiovascular and gastrointestinal health impairments resulting from gambling, health care costs related to treating the consequences of physical violence, and municipalities’ costs of homelessness services. Among indirect costs, productivity loss from temporary and permanent sick leaves could not be included. For intangible costs, the main limitation was the lack of a generally accepted valuation method. Non-Swedish data input were used for the crime rate and the workplace productivity. The scenario analysis in the online appendix (Additional file 2: Appendix figure A1) shows that these parameters only marginally affect the main results.

## Conclusions

Gambling problems are beginning to be recognized as a public health issue. This study found that the costs of problem gambling to society are high and amounted to €1.42 billion in Sweden in 2018, corresponding to around €139 per inhabitant. Even compared to other preventable and more common lifestyle factors such as smoking and alcohol consumption, the societal costs of problem gambling are not negligible.

Most of the costs of problem gambling arise indirectly and relate to unemployment, reduced workplace productivity, and suicide. Intangible costs relating to physical violence and emotional distress experienced by the gambler and other household members are also of significant size. Costs for prevention and treatment are tiny in comparison. Recent law changes oblige regional health care providers and local social care providers in Sweden to invest more in prevention and treatment of problem gambling. This will increase the costs associated with these measures. If these measures prove to be effective, they might help to reduce the societal costs in the future through a reduction of indirect and intangible costs.

## Supplementary Information

**Additional file 1: Appendix Table A1.** Characteristics of the study population. Socio-demographic, psycho-social and clinical problem variables of the study population. * = shares so small (or negative) that they are assumed to be equal to zero. Poor mental health corresponds to 5–24 points on the Kessler Psychological Distress Scale (K6). Experiences of divorce and separation refer to the past 12 months. Unemployment and employment refer to a person’s main employment status during the past 12 months. Employment covers employed and self-employed people. The number of people aged 16–74 in the four groups are 6,908,600, 220,600, 55,600, and 40,500, respectively.

**Additional file 2: Appendix Figure A1.** Scenario analysis of the societal costs of problem gambling. CI = confidence interval. The upper value shows the societal costs if the input parameter is increased, and the lower value if the input parameter is decreased.

## Data Availability

All data analyzed during this study are included in this published article and its supplementary information files. Data derived from the Swelogs general population study can be made available after contact with the Swedish Public Health Agency, which holds a study database accessible from within the European Union.

## Abbreviations

APA DSM: American Psychiatric Association’s Diagnostic and Statistical Manual of Mental Disorders
AUD: Australian dollars
CBT: Cognitive behavioral therapy
COI: Cost of illness
CSOs: Concerned significant others
GDP: Gross domestic product
IPV: Intimate partner violence
PGSI: Problem Gambling Severity Index
Swelogs: Swedish Longitudinal Gambling Study

## References

[1] Calado F, Griffiths MD (2016). Problem gambling worldwide: an update and systematic review of empirical research (2000-2015). J Behav Addict.

[2] Griffiths M (2009). Problem gambling in Europe: an overview.

[3] The Public Health Agency of Sweden. Swedish longitudinal gambling study (Swelogs). Available from: https://www.folkhalsomyndigheten.se/livsvillkor-levnadsvanor/alkohol-narkotika-dopning-tobak-och-spel-andts/spel/swelogs-befolkningsstudie/ (Accessed 5 May 2020].

[4] Abbott M, Binde P, Clark L, Hodgins D, Korn D, Pereira A (2015). Conceptual Framework of Harmful Gambling: An International Collaboration (Revised Edition).

[5] Langham E, Thorne H, Browne M, Donaldson P, Rose J, Rockloff M (2016). Understanding gambling related harm: a proposed definition, conceptual framework, and taxonomy of harms. BMC Public Health.

[6] Dowling NA, Cowlishaw S, Jackson AC, Merkouris SS, Francis KL, Christensen DR (2015). The prevalence of comorbid personality disorders in treatment-seeking problem gamblers: a systematic review and meta-analysis. J Personal Disord.

[7] Lorains FK, Cowlishaw S, Thomas SA (2011). Prevalence of comorbid disorders in problem and pathological gambling: systematic review and meta-analysis of population surveys. Addiction..

[8] Salonen AH, Hellman M, Latvala T, Castrén S (2018). Gambling participation, gambling habits, gambling-related harm, and opinions on gambling advertising in Finland in 2016. Nordic Stud Alcohol Drugs.

[9] Binde P (2013). Why people gamble: a model with five motivational dimensions. Int Gambl Stud.

[10] Schull ND (2002). Escape mechanism: women, caretaking, and compulsive machine gambling.

[11] Weatherly JN (2013). The relationship between endorsing gambling as an escape and the display of gambling problems. J Addict.

[12] Hakansson A, Karlsson A, Widinghoff C (2018). Primary and secondary diagnoses of gambling disorder and psychiatric comorbidity in the Swedish health care system-a Nationwide register study. Front Psychiatry.

[13] Karlsson A, Hakansson A (2018). Gambling disorder, increased mortality, suicidality, and associated comorbidity: a longitudinal nationwide register study. J Behav Addict.

[14] Revheim T, Buvik K (2009). Opportunity structure for gambling and problem gambling among employees in the transport industry. Int J Ment Heal Addict.

[15] Binde P (2016). Preventing and responding to gambling-related harm and crime in the workplace. Nordic Stud Alcohol Drugs.

[16] Binde P. Gambling-related employee embezzlement: a study of Swedish newspaper reports. J Gambling Issues. 2016;34:12-31.

[17] Kourgiantakis T, Saint-Jacques MC, Tremblay J (2013). Problem gambling and families: a systematic review. J Soc Work Pract Addict.

[18] Riley BJ, Harvey P, Crisp BR, Battersby M, Lawn S. Gambling-related harm as reported by concerned significant others: a systematic review and meta-synthesis of empirical studies. J Fam Stud. 2018. Advance online publication. 10.1080/13229400.2018.1513856.

[19] Dowling N, Suomi A, Jackson A, Lavis T, Patford J, Cockman S (2016). Problem gambling and intimate partner violence: a systematic review and meta-analysis. Trauma Violence Abuse.

[20] Svensson J, Romild U, Shepherdson E (2013). The concerned significant others of people with gambling problems in a national representative sample in Sweden - a 1 year follow-up study. BMC Public Health.

[21] Rosenthal RJ (2020). Inclusion of pathological gambling in DSM-III, its classification as a disorder of impulse control, and the role of Robert Custer. Int Gambl Stud.

[22] American Psychiatric Association (2013). Diagnostic and statistical manual of mental disorders (5th ed.).

[23] Abbott M (2017). The epidemiology and impact of gambling disorder and other gambling-related harm. Discussion paper.

[24] World Health Organization (2003). WHO Framework Convention on Tobacco Control.

[25] European Commission (2014). Commission Recommendation of 14 July 2014 on principles for the protection of consumers and players of online gambling services and for the prevention of minors from gambling online. Commission staff working document.

[26] Productivity Commission (1999). Australia’s Gambling Industries. Report No 10.

[27] Productivity Commission. Gambling. Report No 50. Canberra: Australian Government; 20.

[28] Grinols EL, Kruschwitz RB (2011). The Hidden Social Costs of Gambling. The gambling culture.

[29] Williams RJ, Belanger YD, Arthur JN (2011). Gambling in Alberta: history, current status, and socioeconomic impacts.

[30] Rogers RD, Wardle H, Sharp CA, Wood S, Hughes K, Davies TJ (2019). Gambling as a public health issue in Wales.

[31] Winkler P, Bejdova M, Csemy L, Weissova A (2017). Social costs of gambling in the Czech Republic 2012. J Gambl Stud.

[32] Walker DM (2003). Methodological issues in the social cost of gambling studies. J Gambl Stud.

[33] Walker DM, Barnett AH (1999). The social costs of gambling: an economic perspective. J Gambl Stud.

[34] Byford S, Torgerson DJ, Raftery J (2000). Economic note: cost of illness studies. BMJ..

[35] The Public Health Agency of Sweden. Swelogs. Available from: https://www.folkhalsomyndigheten.se/the-public-health-agency-of-sweden/living-conditions-and-lifestyle/alcohol-narcotics-doping-tobacco-and-gambling/gambling/swelogs/ (Accessed 5 May 2020).

[36] Currie SR, Hodgins DC, Casey DM (2013). Validity of the problem gambling severity index interpretive categories. J Gambl Stud.

[37] Browne M, Greer N, Armstrong T, Doran C, Kinchin I, Langham E (2017). The social cost of gambling to Victoria.

[38] Thorley C, Stirling A, Huynh E (2016). Cards on the table: the cost to government associated with people who are problem gamblers in Britain.

[39] Guinness L, Guinness L, Wiseman V (2011). Counting the costs. Introduction to health economics.

[40] Sculpher MJ, Drummond MF, McGuire A (2001). The role and estimation of productivity costs in economic evaluation. Economic evaluation in health care: merging theory with practice.

[41] Hartunian NS, Smart CN, Thompson MS (1980). The incidence and economic costs of cancer, motor vehicle injuries, coronary heart disease, and stroke: a comparative analysis. Am J Public Health.

[42] Rice DP (1967). Estimating the cost of illness. Am J Public Health Nations Health.

[43] Stockholm County Council (2017). Significantly more women than men are treated for mental illness in the Stockholm County Council [Väsentligt fler kvinnor än män vårdas för psykisk ohälsa i Stockholms läns landsting].

[44] Ekman M, Granstrom O, Omerov S, Jacob J, Landen M (2014). Costs of bipolar disorder, depression, schizophrenia and anxiety [Kostnader för bipolär sjukdom, depression, schizofreni och ångest]. Lakartidningen..

[45] Adolphe A, Khatib L, van Golde C, Gainsbury SM, Blaszczynski A (2019). Crime and gambling disorders: a systematic review. J Gambl Stud.

[46] Andersson E, Toresson Grip E, Norrlid H, Fridhammar A (2017). The societal costs of smoking-related diseases in Sweden [Samhällskostnaden för rökningsrelaterad sjuklighet i Sverige]. IHE rapport 2017:4.

[47] Ramboll Management Consulting (2019). The societal consequences of alcohol - A descriptive socio-economic study [Alkoholens samhällsekonomiska konsekvenser - En beskrivande samhällsekonomisk studie].

[48] Forsström D, Spångberg J, Petterson A, Brolund A, Odeberg J. A systematic review of educational programs and consumer protection measures for gambling: an extension of previous reviews. Addiction Research & Theory. 2020; 10.1080/16066359.2020.1729753.

[49] Ministry of Finance (2018). Gambling Act (2018:1138).

