# Supplementary material for: The societal costs of problem gambling in Sweden
Source: BMC Public Health. 2020 Dec 18;20:1921. doi: 10.1186/s12889-020-10008-9 (PMC7747412; doi:10.1186/s12889-020-10008-9)
Supplement: Supplementary file 1 — Additional file 1: Appendix Table A1. Characteristics of the study population. Socio-demographic, psycho-social and clinical problem variables of the study population. * = shares so small (or negative) that they are assumed to be equal to zero. Poor mental health corresponds to 5–24 points on the Kessler Psychological Distress Scale (K6). Experiences of divorce and separation refer to the past 12 months. Unemployment and employment refer to a person’s main employment status during the past 12 months. Employment covers employed and self-employed people. The number of people aged 16–74 in the four groups are 6,908,600, 220,600, 55,600, and 40,500, respectively. [file 12889_2020_10008_MOESM1_ESM.docx]

Appendix table A1: Characteristics of the study population

|  | Non-problem gamblers | Low-risk gamblers | Moderate-risk gamblers | Problem gamblers |
| --- | --- | --- | --- | --- |
| Number of people | 7,709,000 | 236,000 | 56,000 | 45,000 |
|  |  |  |  |  |
| Poor mental health |  |  |  |  |
| Share of people | 25% | 41% | 56% | 37% |
| Difference compared to non-problem gamblers | - | 16% | 31% | 13% |
|  |  |  |  |  |
| Crime and legal problems |  |  |  |  |
| Share of people who have committed a crime during the past 12 months (Browne et al., 2017) | - | 1.3% | 4.7% | 21.9% |
| Share of crimes leading to a police investigation (29% of row above) | - | 0.4% | 1.4% | 6.4% |
| Share of police investigations resulting in a prosecution in courts (22% of row above) | - | 0.1% | 0.3% | 1.4% |
| Share of court sentences leading to incarceration (10% of row above) | - | 0%* | 0%* | 0.1% |
|  |  |  |  |  |
| Divorce and separation |  |  |  |  |
| Share of people | 5% | 2% | 9% | 24% |
| Difference compared to non-problem gamblers | - | 0%* | 4% | 19% |
|  |  |  |  |  |
| Unemployment (age 16–74) |  |  |  |  |
| Share of people | 2% | 7% | 7% | 40% |
| Difference compared to non-problem gamblers | - | 5% | 5% | 39% |
|  |  |  |  |  |
| Employment (age 16–74) |  |  |  |  |
| Share of people | 60% | 74% | 51% | 40% |
| Share of employed people gambling during working hours | 2% | 13% | 16% | 65% |
| Difference compared to non-problem gamblers | - | 11% | 15% | 64% |
|  |  |  |  |  |
| Physical violence |  |  |  |  |
| Share of people | 3% | 2% | 16% | 16% |
| Difference compared to non-problem gamblers | - | 0%* | 13% | 13% |
