# Supplementary figures and images for: The societal costs of problem gambling in Sweden
Source: BMC Public Health. 2020 Dec 18;20:1921. doi: 10.1186/s12889-020-10008-9 (PMC7747412; doi:10.1186/s12889-020-10008-9)

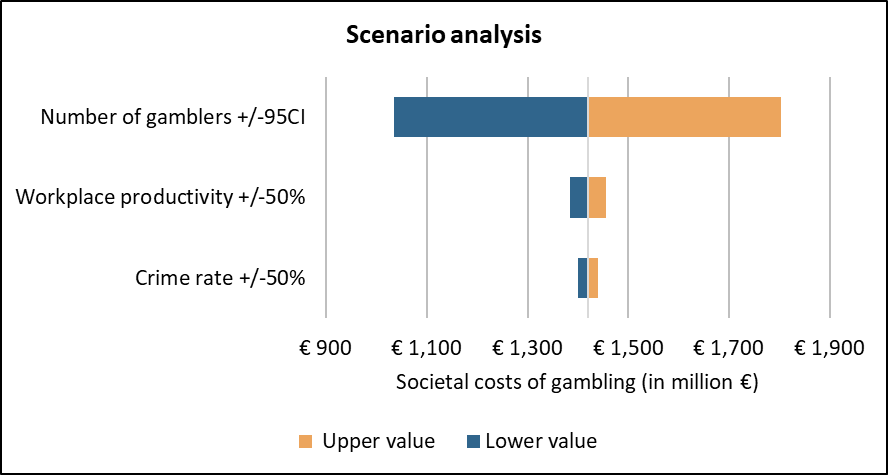


Figure A1: Scenario analysis of the societal costs of problem gambling

Supplement: Supplementary file 2 — Additional file 2: Appendix Figure A1. Scenario analysis of the societal costs of problem gambling. CI = confidence interval. The upper value shows the societal costs if the input parameter is increased, and the lower value if the input parameter is decreased. [file 12889_2020_10008_MOESM2_ESM.docx]
